# A UK nationwide prospective study of treatment change in MODY: genetic subtype and clinical characteristics predict optimal glycaemic control after discontinuing insulin and metformin

**DOI:** 10.1007/s00125-018-4728-6

**Published:** 2018-09-18

**Authors:** Maggie H. Shepherd, Beverley M. Shields, Michelle Hudson, Ewan R. Pearson, Christopher Hyde, Sian Ellard, Andrew T. Hattersley, Kashyap A. Patel, for the UNITED study

**Affiliations:** 1 NIHR Exeter Clinical Research Facility, Royal Devon and Exeter NHS Foundation Trust, Exeter, UK; 2 Institute of Biomedical and Clinical Science, University of Exeter Medical School, RILD, Barrack Road, Exeter, EX2 5DW UK; 3 Division of Population Health and Genomics, School of Medicine, University of Dundee, Dundee, UK; 4 Exeter Test Group, Institute of Health Research, University of Exeter Medical School, University of Exeter, Exeter, UK; 5 Department of Molecular Genetics, Royal Devon and Exeter NHS Foundation Trust, Exeter, UK

**Keywords:** Genetic testing, Glucokinase, Hepatocyte nuclear factor 1α, Hepatocyte nuclear factor 4α, Maturity onset diabetes of the young, Sulfonylurea, Treatment change

## Abstract

**Aims/hypothesis:**

Treatment change following a genetic diagnosis of MODY is frequently indicated, but little is known about the factors predicting future treatment success. We therefore conducted the first prospective study to determine the impact of a genetic diagnosis on individuals with *GCK*-, *HNF1A*- or *HNF4A*-MODY in the UK, and to identify clinical characteristics predicting treatment success (i.e. HbA_1c_ ≤58 mmol/mol [≤7.5%]) with the recommended treatment at 2 years.

**Methods:**

This was an observational, prospective, non-selective study of individuals referred to the Exeter Molecular Genetic Laboratory for genetic testing from December 2010 to December 2012. Individuals from the UK with *GCK*- or *HNF1A/HNF4A*-MODY who were not on recommended treatment at the time of genetic diagnosis, and who were diagnosed below the age of 30 years and were currently aged less than 50 years, were eligible to participate.

**Results:**

A total of 44 of 58 individuals (75.9%) changed treatment following their genetic diagnosis. Eight individuals diagnosed with *GCK*-MODY stopped all diabetes medication without experiencing any change in HbA_1c_ (49.5 mmol/mol [6.6%] both before the genetic diagnosis and at a median of 1.25 years’ follow-up without treatment, *p* = 0.88). A total of 36 of 49 individuals (73.5%) diagnosed with *HNF1A/HNF4A*-MODY changed treatment; however, of the 21 of these individuals who were being managed with diet or sulfonylurea alone at 2 years, only 13 (36.1% of the population that changed treatment) had an HbA_1c_ ≤58 mmol/mol (≤7.5%). These individuals had a shorter diabetes duration (median 4.6 vs 18.1 years), lower HbA_1c_ (58 vs 73 mmol/mol [7.5% vs 8.8%]) and lower BMI (median 24.2 vs 26.0 kg/m^2^) at the time of genetic diagnosis, compared with individuals (*n* = 23/36) with an HbA_1c_ >58 mmol/mol (>7.5%) (or <58 mmol/mol [<7.5%] on additional treatment) at the 2 year follow-up. Overall, 64% (7/11) individuals with a diabetes duration of ≤11 years and an HbA_1c_ of ≤69 mmol/mol (≤8.5%) at time of the genetic test achieved good glycaemic control (HbA_1c_ ≤58 mmol/mol [≤7.5%]) with diet or sulfonylurea alone at 2 years, compared with no participants with a diabetes duration of >11 years and an HbA_1c_ of >69 mmol/mol (>8.5%) at the time of genetic diagnosis.

**Conclusions/interpretation:**

In participants with *GCK*-MODY, treatment cessation was universally successful, with no change in HbA_1c_ at follow-up. In those with *HNF1A/HNF4A*-MODY, a shorter diabetes duration, lower HbA_1c_ and lower BMI at genetic diagnosis predicted successful treatment with sulfonylurea/diet alone, supporting the need for early genetic diagnosis and treatment change. Our study suggests that, in individuals with *HNF1A/HNF4A*-MODY with a longer duration of diabetes (>11 years) at time of genetic test, rather than ceasing current treatment, a sulfonylurea should be added to existing therapy, particularly in those who are overweight or obese and have a high HbA_1c_.

**Electronic supplementary material:**

The online version of this article (10.1007/s00125-018-4728-6) contains peer-reviewed but unedited supplementary material, which is available to authorised users.

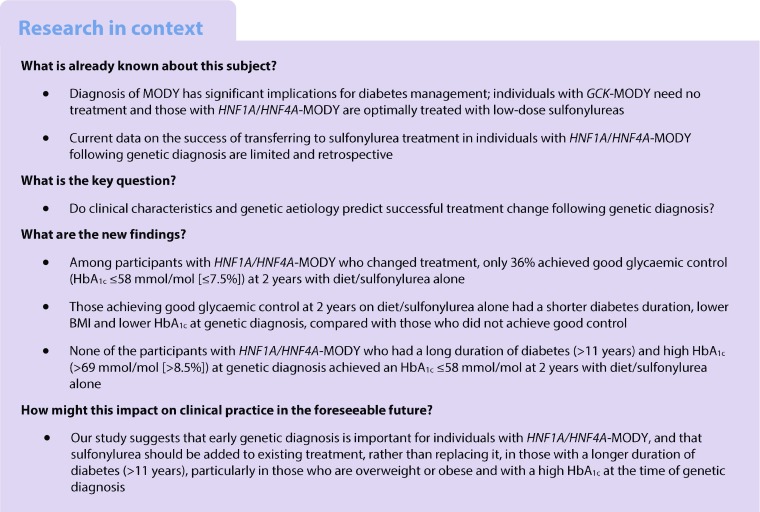



## Introduction

In the UK, MODY accounts for 3.6% of diabetes cases in individuals diagnosed younger than 30 years [1]. Diagnosis of MODY has significant implications for diabetes management. *GCK*-MODY causes asymptomatic, mild fasting hyperglycaemia (usually 5.4–8.3 mmol/l) [5]. The glucose level is regulated at a higher level in *GCK*-MODY, making glucose-lowering treatment ineffective [6], and therefore treatment is not recommended [4]. Individuals with *HNF1A*- or *HNF4A*-MODY are optimally treated with low-dose sulfonylureas [7–10] because of an increased pancreatic insulin secretory response to sulfonylureas and increased insulin sensitivity to the insulin secreted [7].

There is a significant delay from the diagnosis of diabetes to the correct molecular genetic diagnosis of MODY [2, 11–14]. The majority of individuals are initially misdiagnosed with type 1 or type 2 diabetes and inappropriately treated [12, 15–20].

Current data on the success of transfer to sulfonylurea treatment in individuals with *HNF1A*/*HNF4A*-MODY following genetic diagnosis are limited and retrospective [11, 21]. In one case, a study focused on individuals in a single centre with expertise in monogenic diabetes [10]. There have been no prospective studies that have assessed the success of treatment change, glycaemic control and maintenance on recommended treatment following genetic diagnosis in individuals with MODY in non-specialist centres.

The aims of our study were to determine the impact of a genetic diagnosis on diabetes treatment in UK individuals with *GCK*-, *HNF1A*- or *HNF4A*-MODY, and to identify clinical characteristics that predict successful management (i.e. HbA_1c_ ≤58 mmol/mol [≤7.5%]) with no treatment in those with *GCK*-MODY or sulfonylureas in those with *HNF1A* and *HNF4A*-MODY at 2 years after genetic diagnosis.

## Methods

### Study design

This was an observational, prospective, non-selective study of all individuals with *HNF1A*/*HNF4A*- or *GCK*-MODY identified from routine UK referrals to the Exeter Molecular Genetic Laboratory for genetic testing from December 2010 to December 2012. Ethics approval was granted by the NRES Committee South West–Central Bristol (REC no. 10/H0106/03). This study was part of the UNITED (Using pharmacogeNetics to Improve Treatment in Early-onset Diabetes) study which aimed to determine prevalence of monogenic diabetes in those diagnosed with diabetes below the age of 30 years [1]. All study participants gave informed consent (with parental consent and children’s assent gained for those younger than 16 years, *n* = 9).

### Individual characteristics

Individuals were eligible to participate if: (1) genetic testing confirmed *HNF1A*-, *HNF4A*- or *GCK*-MODY; (2) they were not on recommended treatment at time of genetic diagnosis; and (3) they had been diagnosed with diabetes when younger than 30 years and were younger than 50 years at time of genetic testing. Treatment was considered ‘non-recommended’ if those with *HNF1A*/*HNF4A*-MODY were treated with medication other than sulfonylureas and those with *GCK*-MODY were taking any diabetes therapy.

Overall, 305 individuals referred from across the UK were confirmed to have *GCK*-MODY (*n* = 112), *HNF1A*-MODY (*n* = 143) or *HNF4A*-MODY (*n* = 50) within the duration of this study. A total of 244 individuals did not meet eligibility criteria and were not followed up: 101 were excluded on age criteria (37 with *GCK*-MODY, 43 with *HNF1A*-MODY and 21 with *HNF4A*-MODY) and 143 were excluded based on treatment criteria (63 with *GCK*-MODY, 61 with *HNF1A*-MODY and 19 with *HNF4A*-MODY).

**Fig. 1:**
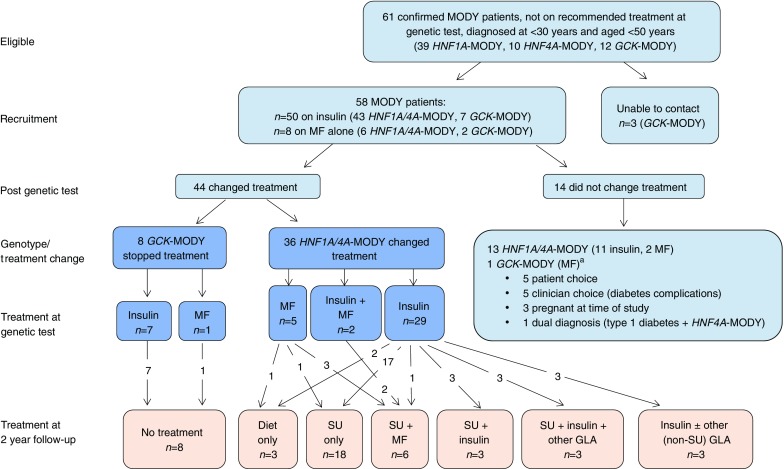
Flow chart indicating recruitment, treatment at genetic diagnosis and treatment at 2 years after the genetic diagnosis. GLA, glucose-lowering agent; MF, metformin; SU, sulfonylurea. ^a^This individual with *GCK*-MODY was initially treated with insulin and metformin but did not stop all treatment following the genetic test

Therefore, 61 individuals fulfilled the eligibility criteria. Of these, 58 were contactable and agreed to participate (39 with *HNF1A*-MODY, 10 with *HNF4A*-MODY and nine with *GCK*-MODY; Fig. 1). This included 11 related individuals from five families: two parent–child pairs with *HNF1A*-MODY; one family in which the mother, her son and her sister had *HNF1A*-MODY; one sibling pair with *HNF1A*-MODY; and one sibling pair with *GCK*-MODY (see electronic supplementary material [ESM] Table 1). All the individuals in the study were white, except one who was of mixed white and East Asian ethnicity. There were 41 women. The median age at the diagnosis of diabetes was 17 (interquartile range [IQR] 13–21] years; at the time of genetic testing median BMI was 24.8 (IQR 21.9–28.2) kg/m^2^, duration of diabetes 10 (IQR 2–20) years and baseline HbA_1c_ 59.5 (IQR 50–73) mmol/mol (7.6% [IQR 6.7–8.8%]). At the time of genetic diagnosis, 50 individuals (86.2%) were being treated with insulin (43 with *HNF1A*/*HNF4A*-MODY and seven with *GCK*-MODY) and eight (13.8%) were taking metformin alone (six with *HNF1A*/*HNF4A*-MODY and two with *GCK*-MODY). Of those on insulin, 46 were on insulin alone and four took metformin in addition. The BMI for children under the age of 19 years was adjusted to the adult equivalent using the Child Growth Foundation Reference Standards [22].

### Follow-up and treatment

Individuals were telephoned at baseline (i.e. the time of the genetic test result) and at 3, 6, 12 and 24 months. Self-reported diabetes treatment was recorded. HbA_1c_ was measured at baseline (prior to treatment change) and at 3, 6 and 12 months from ‘finger-prick’ blood samples that were collected at home and posted to the Blood Sciences laboratory at the Royal Devon and Exeter NHS Foundation Trust. HbA_1c_ results at 24 months were accessed from the individual’s local laboratory. Genetic reports included a statement indicating the recommended treatment for *GCK*-, *HNF1A*- and *HNF4A*-MODY, but all decisions regarding diabetes management after genetic diagnosis were made by local clinicians.

### Statistical analysis

Non-parametric tests (Mann–Whitney test for continuous variables, *χ*^2^ or Fisher’s exact test for categorical variables) were used to compare the characteristics of treatment groups. The Wilcoxon matched-pairs signed-ranks test was used to compare HbA_1c_ results before and after the genetic diagnosis. Continuous data are expressed as medians (IQR). A *p* value <0.05 was considered significant. For two individuals, a single HbA_1c_ value was imputed assuming a linear trend between two available HbA_1c_ points. Analysis was conducted using Stata/SE 14 (StataCorp, College Station, TX, USA).

## Results

A total of 44 of 58 individuals (75.9%) changed treatment following the genetic diagnosis (Fig. 1, ESM Table 1). Eleven of the 44 participants (25%) were younger than 18 years (four with *GCK*-MODY, six with *HNF1A*-MODY and one with *HNF4A*-MODY) at the time of genetic diagnosis. Fourteen individuals (24.1%) did not change treatment and were not followed-up. Reasons for continuing with the previous treatment were pregnancy (*n =* 3), individual choice (*n* = 5) and clinician choice (*n* = 5); this included individuals with retinopathy and nephropathy or concomitant confirmed type 1 diabetes (*n* = 1, GAD-antibody positive, urine C-peptide creatinine ratio 0.12 nmol/mol) (Fig. 1).

Eight of nine individuals with *GCK*-MODY (including seven previously treated with insulin) stopped all diabetes treatment following their genetic diagnosis, irrespective of diabetes duration (median 1.8 [IQR 0.6–7.2] years) and BMI (median 19.8 [IQR 17.9–22.7] kg/m^2^). HbA_1c_ remained the same at a median of 1.25 (IQR 1–2) years’ follow-up without any treatment (49.5 [IQR 47–52] mmol/mol [6.6%, IQR 6.4–6.9%] at the genetic diagnosis vs 49.5 [IQR 47–50.5] mmol/mol [6.6%, IQR 6.5–6.8%] at follow-up, *p* = 0.88) (ESM Fig. 1). One individual with *GCK*-MODY stopped insulin but remained on metformin through the clinician’s choice; however, all recorded HbA_1c_ values were 52–57 mmol/mol (6.9–7.4%), which are consistent with levels seen in *GCK*-MODY.

A total of 36 of 49 (73.5%) individuals diagnosed with *HNF1A*/*HNF4A*-MODY changed treatment following the genetic diagnosis (Fig. 1). Of these, 21 of 36 (58%) were treated with diet (*n* = 3) or sulfonylurea (*n* = 18) alone at 2 years. Thirteen of these 21 individuals (62%) had HbA_1c_ ≤58 mmol/mol (≤7.5%) at 2 years (Table 1).

**Table 1:** Characteristics of individuals with *HNF1A/HNF4A*-MODY at genetic diagnosis and at 2 year follow-up

Characteristic	HbA_1c_ ≤58 mmol/mol (≤7.5%) on diet/sulfonylurea alone at 2 years (*n* = 13)	HbA_1c_ >58 or ≤58 mmol/mol (>7.5 or ≤7.5%) on additional treatment at 2 years (*n* = 23)	*p* value
At genetic diagnosis/treatment transfer			
Age at diabetes diagnosis, years	18.3 (14.9–21.5)	16.3 (12.8–19.1)	0.18
Duration of diabetes, years	4.6 (1.0–8.1)	18.1 (4.0–24.9)	0.01
BMI, kg/m^2^	24.2 (21.7–25.3)	26.0 (24.9–30.9)	0.02
HbA_1c_, mmol/mol	58 (52–60)	73 (62–86)	0.005
HbA_1c,_ %	7.5 (6.9–7.6)	8.8 (7.8–10)	
Women	9 (69)	19 (83)	0.42
Treatment			0.52
Insulin	12 (92)	17 (74)	
Insulin + metformin	0	2 (9)	
Metformin	1 (8)	4 (17)	
Genetic aetiology			1
*HNF1A*	11 (85)	18 (79)	
*HNF4A*	2 (15)	5 (21)	
At 2 year follow-up			
HbA_1c_, mmol/mol	46 (43–55)	77 (67–86)	<0.001
HbA_1c,_ %	6.4 (6.1–7.2)	9.2 (8.3–10.0)	
HbA_1c_ <58 mmol/mol (<7.5%)	13 (100)	1 (4)	
Treatment			
Diet	1 (8)	2 (9)	
Sulfonylurea	12 (92)	6 (26)	
Sulfonylurea + metformin	0	6 (26)	
Sulfonylurea + insulin	0	3 (13)	
Sulfonylurea + insulin + other GLA	0	3 (13)	
Insulin ± non-sulfonylurea GLA	0	3 (13)	

Data are median (IQR) for continuous variables and *n* (%) for categorical variables

GLA, glucose-lowering agent

We next compared the clinical characteristics of the 13 individuals (36.1%, 13/36) with *HNF1A*/*HNF4A*-MODY being managed with sulfonylurea/diet alone who achieved an HbA_1c_ ≤58 mmol/mol (≤7.5%) with those of the 23 individuals with an HbA_1c_ >58 mmol/mol (>7.5%) (*n* = 22) or ≤58 mmol/mol (≤7.5%) on additional treatment (*n* = 1) at 2 year follow-up (Table 1). The individuals with an HbA_1c_ ≤58 mmol/mol (≤7.5%) on sulfonylurea/diet alone at 2 years had a shorter diabetes duration (median 4.6 vs 18.1 years), lower BMI (median 24.2 vs 26.0 kg/m^2^) and lower HbA_1c_ (58 vs 73 mmol/mol [7.5 vs 8.8%]) at treatment transfer compared with those with an HbA_1c_ >58 mmol/mol (>7.5%) or the single individual with an HbA_1c_ <58 mmol/mol (<7.5%) on additional treatment (Table 1). There was no difference in genetic aetiology between these groups (ESM Table 1). Those managed with sulfonylurea/diet alone at 2 years improved their HbA_1c_ from a median of 58 mmol/mol (7.5%) pre-genetic diagnosis to 46 mmol/mol (6.4%) at 2 years (*p* = 0.001). This contrasted with the other group, in which HbA_1c_ increased (median 73 vs 77 mmol/mol [8.8 vs 9.2%]), *p* = 0.03) (Table 1). Individuals in the latter group who were taking sulfonylureas were on maximum recommended dose (gliclazide 160 mg twice daily).

**Fig. 2:**
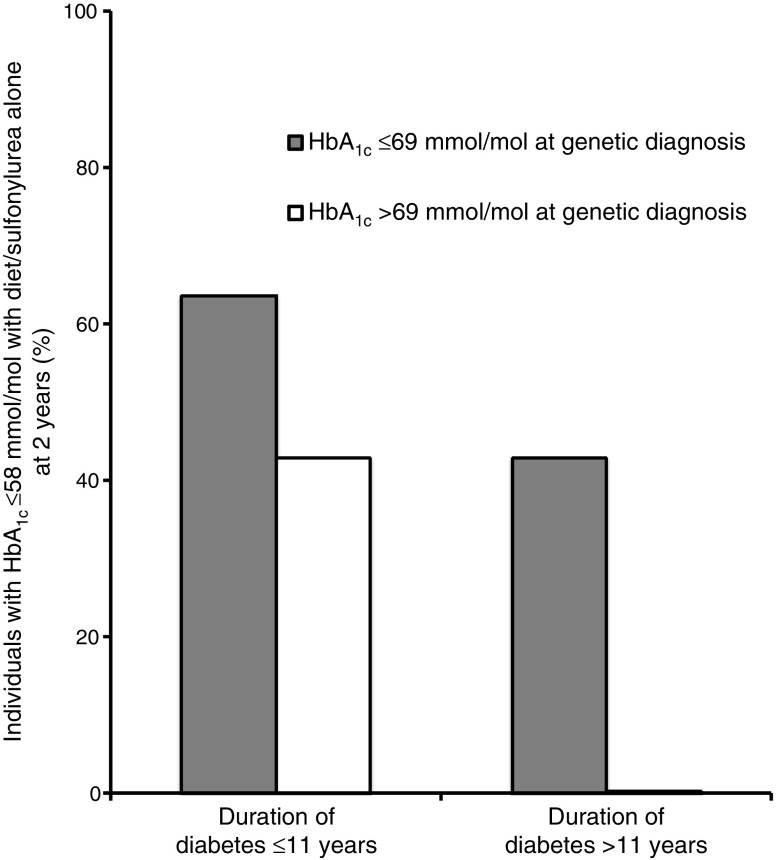
Effect of duration of diabetes and HbA_1c_ at genetic diagnosis on the ability to achieve good glycaemic control with diet/sulfonylurea alone at 2 years following genetic diagnosis in individuals with *HNF1A/HNF4A*-MODY. Participants were divided into groups according to HbA_1c_ (≤69 or >69 mmol/mol [≤8.5% or >8.5%], *n* = 18 in each group) and duration of diabetes (≤11 or >11 years, *n* = 18 in each group) using the median values of the *HNF1A/HNF4A*-MODY cohort (*n* = 36). The participant numbers in each of the groups were: HbA_1c_ ≤69 mmol/mol (≤8.5%) and duration ≤11 years, *n* = 11; HbA_1c_ ≤69 mmol/mol (≤8.5%) and duration >11 years, *n* = 7; HbA_1c_ >69 mmol/mol (>8.5%) and duration ≤11 years, *n* = 7; and HbA_1c_ >69 mmol/mol (>8.5%) and duration >11 years, *n* = 11. The number of individuals who had an HbA_1c_ ≤58 mmol/mol (≤7.5%) with diet or sulfonylurea alone at 2 years in these four groups was *n* = 7, *n* = 3, *n* = 3 and *n* = 0, respectively

We also assessed the combined effect of diabetes duration and HbA_1c_ at genetic diagnosis on the ability to achieve good glycaemic control with diet/sulfonylurea alone in individuals with *HNF1A/HNF4A*-MODY. We divided the cohort by median diabetes duration (≤11 vs >11 years) and median HbA_1c_ (≤69 vs >69 mmol/mol [≤8.5% vs >8.5%]) at genetic diagnosis (Fig. 2). A total of 10/18 individuals (56%) with a shorter diabetes duration achieved optimal control, compared with 3/18 (17%) with longer diabetes duration (*p* = 0.03). Similarly, 10/18 (56%) with lower HbA_1c_ at genetic diagnosis achieved optimal control compared with 3/18 (17%) with higher HbA_1c_ at genetic diagnosis (*p* = 0.03). A total of 7 of 11 individuals (64%) with shorter diabetes duration and lower HbA_1c_ at genetic diagnosis achieved optimal control, while none of the individuals (0/11) with longer duration and higher HbA_1c_ at genetic diagnosis achieved an HbA_1c_ ≤58 mmol/mol (≤7.5%) with diet/sulfonylurea alone (*p* = 0.02). Similar results were seen for diabetes duration and BMI at genetic diagnosis (ESM Fig. 2).

## Discussion

This national, prospective, non-selective study demonstrates that most individuals with MODY commence the recommended treatment after a genetic diagnosis has been confirmed. However, only 58% of individuals with *HNF1A/HNF4A*-MODY were on diet or sulfonylurea alone at 2 years and, overall, just 36% of individuals with *HNF1A/HNF4A*-MODY who changed treatment achieved the good glycaemic control (≤58 mmol/mol [≤7.5%]) needed to avoid diabetes complications. Our study suggests that successful treatment with diet/sulfonylurea alone was most likely in those with *HNF1A/HNF4A*-MODY who had a shorter duration of diabetes, healthy BMI and lower HbA_1c_ at the time of genetic diagnosis. All participants with *GCK*-MODY were able to stop insulin or oral hypoglycaemic agents without deterioration in glycaemic control, as previously shown [6]. Identifying those with *GCK*-MODY is important, as all diabetes treatments can be discontinued and follow-up is not required [2–4, 6].

Improvement in glycaemic control among individuals with *HNF1A*/*HNF4A*-MODY is needed to prevent diabetes complications. Individuals with *HNF1A/HNF4A*-MODY are at increased, or at least the same, risk of developing diabetes-related complications compared with those with other diabetes subtypes [23, 24]. Our study showed that despite transfer to the recommended treatment, only 36% of individuals achieved an HbA_1c_ ≤58 mmol/mol (≤7.5%) on sulfonylurea/diet alone. The lack of optimal glycaemic control in our study may have resulted from clinical inertia or limited experience among local clinicians in managing *HNF1A/HNF4A*-MODY and previous advice advocating a trial of sulfonylureas even in those with longstanding diabetes [11]. The lack of standardised treatment guidelines for individuals needing additional second-line therapy may also contribute to suboptimal glycaemic control. Our results are similar, albeit lower, than those of previous studies, which found that around 50–62% of participants attained an HbA_1c_ ≤58 mmol/mol (≤7.5%) with sulfonylurea therapy alone [10, 11]. The difference in the results may be a result of differences in the duration of diabetes at genetic diagnosis.

Progressive loss of pancreatic beta cell function is a feature of *HNF1A*/*HNF4A*-MODY, resulting in increasing glycaemia and increasing treatment requirements over time [25]. Successful treatment change and achieving good glycaemic control is more likely to be achieved if the genetic diagnosis is made early. Prompt transfer to sulfonylureas, enabling optimal glycaemic control soon after diabetes diagnosis, may reduce the risk of future complications in those with *HNF1A/HNF4A*-MODY, as seen with type 1 and type 2 diabetes [26, 27]. If individuals are transferred to optimal treatment early, then it may be easier to achieve good control and to maintain it. This is reflected by our data showing that individuals with lower HbA_1c_ levels at genetic diagnosis are more likely to achieve good glycaemic control at 2 years. In contrast to this, individuals with higher HbA_1c_ levels at genetic diagnosis rarely achieved good glycaemic control with sulfonylureas alone. As a consequence of these data, we now recommend that a sulfonylurea should be added to existing treatment, rather than replacing it, in individuals with *HNF1A*/*HNF4A*-MODY with a longer diabetes duration (>11 years), especially in those with higher HbA_1c_ levels at genetic diagnosis and a BMI >25 kg/m^2^.

In this study, we found that higher HbA_1c_ levels and BMI at genetic diagnosis were associated with reduced success on sulfonylurea treatment in participants with *HNF1A/HNF4A*-MODY. Similar results have been seen in a previous retrospective study [10]. In our data, a higher BMI at genetic diagnosis markedly reduced the success of sulfonylurea therapy in those with a longer duration of diabetes. This is likely to reflect the impact of increased insulin resistance in those with more severe beta cell dysfunction. These data raise the question of whether weight loss may aid glycaemic control in individuals with *HNF1A/HNF4A*-MODY.

Our study has limitations. Treatment decisions were made via local clinicians and were not standardised. We did not collect data regarding changes in BMI over time and any effect this had on treatment requirements, which has previously been shown to negatively affect glycaemic control [10]. It was not appropriate to use multiple regression analysis of factors predicting successful long-term treatment with sulfonylureas alone to identify the relative contribution of each factor because of the small size of our study. We did not measure endogenous insulin secretion at time of genetic diagnosis in our participants and were therefore unable to assess its role in treatment response. Finally, our study did not have a large enough sample size to detect whether specific genetic mutations had an effect on the response to treatment over and above the strongly associated clinical features we identified. Despite these limitations, our study provides the first national prospective data regarding treatment change following genetic diagnosis in non-specialised centres across the UK.

In summary, our national prospective study identified that the majority of individuals changed treatment following a genetic diagnosis of MODY. Those with *GCK*-MODY were able to stop all diabetes treatment with no deterioration in HbA_1c_, highlighting the significance of identifying individuals with *GCK*-MODY as diabetes medication is unnecessary and follow-up is not required. In participants with *HNF1A/HNF4A*-MODY, only 58% were maintained on sulfonylurea/diet alone at 2 years and just 36% of participants with *HNF1A/HNF4A*-MODY who changed treatment achieved an HbA_1c_ ≤58 mmol/mol (≤7.5%) 2 years following genetic diagnosis. A shorter duration of diabetes, lower HbA_1c_ level and lower BMI at genetic diagnosis predicted successful treatment with sulfonylurea/diet alone in participants with *HNF1A/HNF4A*-MODY, supporting the need for early genetic diagnosis and treatment change.

## Electronic supplementary material

ESM (PDF 170 kb)

## Data Availability

The datasets generated and analysed from the study are available from the corresponding author on reasonable request.

## Abbreviation

IQR: Interquartile range
