# Supplementary material for: A UK nationwide prospective study of treatment change in MODY: genetic subtype and clinical characteristics predict optimal glycaemic control after discontinuing insulin and metformin
Source: Diabetologia. 2018 Sep 18;61(12):2520–7. doi: 10.1007/s00125-018-4728-6 (PMC6223847; doi:10.1007/s00125-018-4728-6)
Supplement: Supplementary file 1 — (PDF 170 kb) [file 125_2018_4728_MOESM1_ESM.pdf]

ESM Table 1. Heterozygous MODY causing variants in the *GCK*, *HNF1A* and *HNF4A* genes identified in the study cohort who underwent treatment change (n=44). Variants are described according to HGVS nomenclature guidelines (http://varnomen.hgvs.org/) using reference sequences NM_000162.4 for *GCK*, NM_000545.6 for *HNF1A* and NM_175914.4 for *HNF4A*. 0 in Glycaemic control column means HbA1c ≤58mmol/mol (≤7.5%) with Diet/SU alone at 2 years and 1 means HbA_{1c} >58 or ≤58mmol/mol (>7.5 or ≤7.5%) on additional treatment at 2 years.

studyId	Relationship	Gene	Protein Change	Nucleotide Change	Protein Effect	Variant Classification	Glycaemic control
94		*GCK*	p.(Ser340Ile)	c.1019G>T	Missense	Pathogenic	
360		*GCK*	p.(Val253fs)	c.757del	Frameshift	Pathogenic	
624	Proband	*GCK*	p.(Val374fs)	c.1119_1120dup	Frameshift	Pathogenic	
625	Sister of 624	*GCK*	p.(Val374fs)	c.1119_1120dup	Frameshift	Pathogenic	
1025		*GCK*	p.(Tyr215Ter)	c.645C>A	Stop gain	Pathogenic	
1194		*GCK*	p.(Arg191Trp)	c.571C>T	Missense	Pathogenic	
1375		*GCK*	p.(Lys39del)	c.115_117del	In-frame deletion	Likely Pathogenic	
1406		*GCK*	p.(Phe150Ser)	c.449T>C	Missense	Pathogenic	
110		*HNF1A*	p.(Glu275del)	c.824_826del	In-frame deletion	Pathogenic	0
227	Sister of 351	*HNF1A*	p.(Gly207Asp)	c.620G>A	Missense	Pathogenic	1
228	Son of 351	*HNF1A*	p.(Gly207Asp)	c.620G>A	Missense	Pathogenic	0
351	Proband	*HNF1A*	p.(Gly207Asp)	c.620G>A	Missense	Pathogenic	1
361		*HNF1A*	p.(Gly292fs)	c.872dup	Frameshift	Pathogenic	1
394	Mother of 395	*HNF1A*	p.(Pro291fs)	c.872del	Frameshift	Pathogenic	1
395	Proband	*HNF1A*	p.(Pro291fs)	c.872del	Frameshift	Pathogenic	1
455		*HNF1A*	p.(Gly292fs)	c.872dup	Frameshift	Pathogenic	0
529		*HNF1A*	p.(Thr260Met)	c.779C>T	Missense	Pathogenic	0
599		*HNF1A*	p.(Arg203His)	c.608G>A	Missense	Pathogenic	0
618		*HNF1A*	p.(Asn62fs)	c.185del	Frameshift	Pathogenic	1
620		*HNF1A*	p.(Pro291fs)	c.872del	Frameshift	Pathogenic	0
659		*HNF1A*	p.(Pro291fs)	c.872del	Frameshift	Pathogenic	0
682		*HNF1A*	p.?	c.1502-6G>A	Aberrant splicing	Pathogenic	1
756		*HNF1A*	p.?	c.714-2A>G	Aberrant splicing	Pathogenic	1
771	Sister of 772	*HNF1A*	p.(Gly292fs)	c.872dup	Frameshift	Pathogenic	1
772	Sister of 771	*HNF1A*	p.(Gly292fs)	c.872dup	Frameshift	Pathogenic	1
793		*HNF1A*	p.(Arg203His)	c.608G>A	Missense	Pathogenic	1
867		*HNF1A*	p.(Ser608fs)	c.1822_1823del	Frameshift	Pathogenic	1
897	Mother of 900	*HNF1A*	p.(Pro447Leu)	c.1340C>T	Missense	Pathogenic	1
900	Proband	*HNF1A*	p.(Pro447Leu)	c.1340C>T	Missense	Pathogenic	1
1022		*HNF1A*	p.(Gly292fs)	c.872dup	Frameshift	Pathogenic	1
1067		*HNF1A*	p.(Ala276Asp)	c.827C>A	Missense	Likely Pathogenic	0
1178		*HNF1A*	p.(Arg271Gln)	c.812G>A	Missense	Pathogenic	1
1331		*HNF1A*	p.?	c.956-2A>C	Aberrant splicing	Pathogenic	0
1357		*HNF1A*	p.(Pro379Thr)	c.1135C>A	Missense	Likely	1

						Pathogenic	
8000089		*HNF1A*	p.(Asn450fs)	c.1349dup	Frameshift	Pathogenic	1
8000170		*HNF1A*	p.(Arg131Trp)	c.391C>T	Missense	Pathogenic	0
8002003		*HNF1A*	p.(Thr10Pro)	c.28A>C	Missense	Likely Pathogenic	0
894		*HNF4A*	p.(Ala107Asp)	c.320C>A	Missense	Pathogenic	1
917		*HNF4A*	p.(Leu247Pro)	c.740T>C	Missense	Pathogenic	1
1085		*HNF4A*	p.(Arg112Gln)	c.335G>A	Missense	Pathogenic	0
1117		*HNF4A*	p.(Arg114Trp)	c.340C>T	Missense	Pathogenic	1
1339		*HNF4A*	p.(Arg290His)	c.869G>A	Missense	Pathogenic	1
1348		*HNF4A*	p.(Arg114Trp)	c.340C>T	Missense	Pathogenic	1
1380		*HNF4A*	p.?	c.319+5G>A	Aberrant splicing	Likely Pathogenic	0

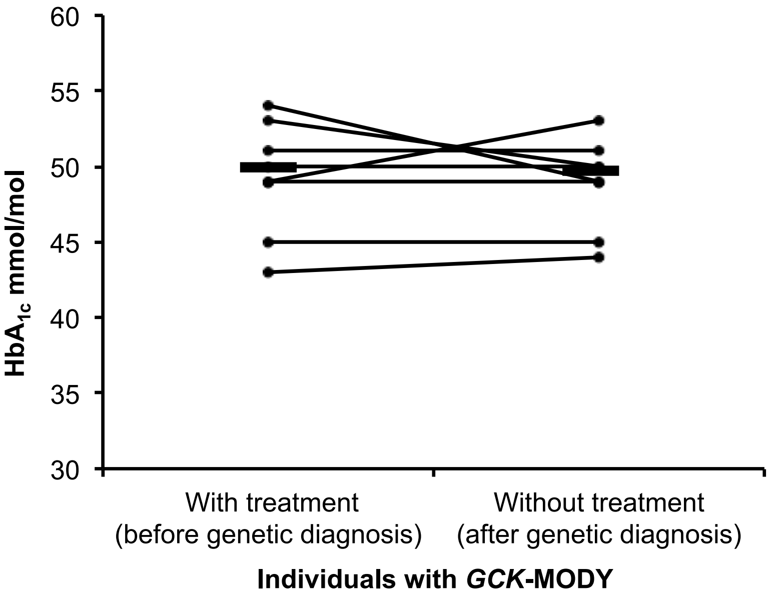

ESM Fig 1: HbA_{1c} of individuals with *GCK*-MODY before and after the post genetic diagnosis. The graph shows the HbA_{1c} for the individuals with *GCK*-MODY (*n*=8) before the genetic diagnosis on treatment and after stopping the treatment for median 1.25 years (IQR 1-2 , range 0.25 to 3 years) following the genetic diagnosis. Median HbA_{1c} is highlighed with black rectangle.

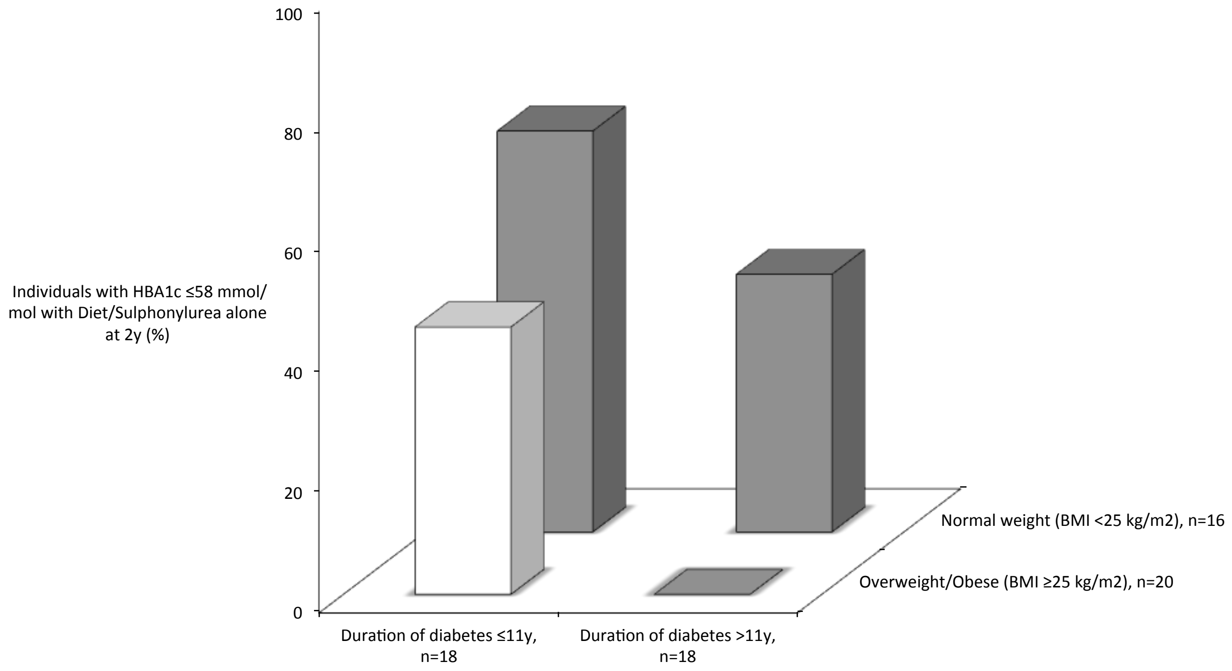

ESM Fig 2. The effect of duration of diabetes and BMI at genetic diagnosis on ability to achieve good glycaemic control with diet/sulfonyurias alone at 2 years post genetic diagnosis in individuals with *HNF1A/HNF4A*-MODY. Duration of diabetes were divided into two groups by the median value of the *HNF1A/HNF4A*-MODY cohort (*n*=36).
